# Perinatal Arterial Ischemic Stroke in Monochorionic Twins: A Retrospective Observational Single-Center Cohort Study

**DOI:** 10.1161/STROKEAHA.125.051702

**Published:** 2025-09-10

**Authors:** Bregje O. van Oldenmark, Mathies Rondagh, Phebe Adama van Scheltema, Femke Slaghekke, Lotte E. van der Meeren, Marjolijn S. Spruijt, Enrico Lopriore, Linda S. de Vries, Sylke. J. Steggerda

**Affiliations:** Division of Neonatology, Department of Pediatrics, Willem-Alexander Children’s Hospital (B.O.v.O., M.R., M.S.S., E.L., L.S.d.V., S.J.S.), Leiden University Medical Center, the Netherlands.; Department of Obstetrics, Gynaecology and Prenatal Diagnostics (P.A.v.S., F.S.), Leiden University Medical Center, the Netherlands.; Department of Pathology (L.E.v.d.M.), Leiden University Medical Center, the Netherlands.; Department of Pathology, Erasmus Medical Center, Rotterdam, the Netherlands (L.E.v.d.M.).; Division of Neonatology, Department of Pediatrics, Radboud Medical Center, Nijmegen, the Netherlands (M.S.S.).

**Keywords:** ischemic stroke, magnetic resonance imaging, placenta, pregnancy, twins

## Abstract

**BACKGROUND:**

Monochorionic twins, characterized by placental sharing and vascular anastomoses, carry a high risk of brain injury, including perinatal arterial ischemic stroke (PAIS). However, the pathophysiology and timing-related risk factors of PAIS remain unclear.

**METHODS:**

Retrospective cohort of all monochorionic twins with neuroimaging-confirmed PAIS born from 2005 to 2024 and evaluated at a Dutch national referral center. PAIS timing was classified as presumed antenatal, direct perinatal, or postnatal onset. Risk factors and neurodevelopmental outcomes, including cerebral palsy and cognitive impairment, were assessed.

**RESULTS:**

Eighteen cases of PAIS were identified among 1183 twin pairs born <35 weeks’ gestation (1.5%). Mean gestational age at birth was 29.4 weeks (95% CI, 28.3–31.5), and mean birth weight was 1258 (95% CI, 1062–1453) grams. Pregnancy complications were identified in 89%: twin-to-twin transfusion syndrome (n=13), twin anemia polycythemia sequence (n=1), and single fetal demise (n=2). In twin-to-twin transfusion syndrome/twin anemia polycythemia sequence, PAIS was diagnosed in the recipient twin in 12 of 14 (86%) cases. Regarding stroke onset, 6 occurred antenatally, 7 direct perinatally, and 5 occurred in the postnatal period. Stroke patterns involved the middle cerebral artery (anterior, posterior, or main branch) in 10 of 18 (56%); perforator arteries arising from middle cerebral artery or anterior cerebral artery in 7 of 18 (38%); anterior cerebral artery in 1 of 18 (6%); and posterior cerebral artery in 1 of 18 (6%). Among 7 infants with main branch middle cerebral artery stroke, 2 died in the fetal or neonatal period, and all 4 with a follow-up assessment developed unilateral spastic cerebral palsy. Among 6 infants with perforator stroke and follow-up, 2 had hemiparesis at 24 months corrected age.

**CONCLUSIONS:**

PAIS in monochorionic twins predominantly affects the recipient twin and can occur in the antenatal, direct perinatal, or postnatal period, with variable stroke patterns and outcomes. We recommend dedicated fetal and postnatal neuroimaging in complicated preterm-born monochorionic twins to detect PAIS and allow early rehabilitation therapy.

Perinatal arterial ischemic stroke (PAIS) is defined in the literature as a focal disruption of cerebral blood flow occurring from the 20th week of gestation through 28 days after birth (at term age).^1,2^ It is a leading cause of unilateral spastic cerebral palsy, as well as cognitive, language, behavioral problems, and epilepsy in childhood.^3^ However, data on preterm infants are limited, as most studies reported to date focus on full-term infants.

The immature cerebral circulation in fetuses and neonates, particularly in preterm infants, makes this population susceptible to PAIS.^4^ Perinatal stroke arises from 3 primary mechanisms: (1) thromboembolism (2) cerebral arteriopathy, and (3) thrombosis.^5,6^ Clinically, PAIS in the full-term infant tends to present with hemiconvulsions or nonspecific symptoms such as altered consciousness, hypotonia, apnea, and feeding difficulties.^7^ In contrast, the preterm infant is usually asymptomatic, resulting in delayed diagnosis until neurological deficits manifest later in life, unless PAIS was diagnosed on routine postnatal imaging.^8^ Identified risk factors for PAIS in the preterm infant include fetal heart rate abnormalities and neonatal hypoglycemia.^9^

PAIS is more prevalent in twin pregnancies compared with singletons, with monochorionic twins being particularly vulnerable.^10–12^ The unique placental angioarchitecture in monochorionic twins predisposes to important complications such as twin-to-twin transfusion syndrome (TTTS), twin anemia polycythemia sequence (TAPS), selective fetal growth restriction, and single fetal demise (sFD).^13^ These complications can lead to brain injury through various mechanisms: placental emboli passing into the fetal cerebral circulation, (iatrogenic) prematurity, hemodynamic imbalances, and perfusion changes during antenatal intervention in TTTS; vascular sludging and vessel obstruction in TAPS recipients; and acute exsanguination in sFD (acute perimortem TTTS).^14,15^ Moreover, most of these twins are born preterm.^16^

Although several hypotheses exist regarding the development of PAIS in monochorionic twins during pregnancy, perinatal and postnatal cases remain poorly understood. We hypothesize that in preterm monochorionic twins, PAIS may occur either before, during, or after birth due to additional obstetric and perinatal risk factors related to monochorionicity. This is different in singletons, in whom stroke is most often related to the immediate perinatal or postnatal period.^9,17^ Therefore, the primary aim of this study was to gain more insight into the time of occurrence of PAIS in monochorionic twins, whether before, during, or after birth. As a secondary aim, these 3 groups were examined for potential risk factors and stroke patterns on fetal and neonatal neuroimaging (cranial ultrasound [cUS] and magnetic resonance imaging [MRI]), the latter with particular focus on the affected vascular territory and involvement of the corticospinal tract. Finally, these findings were correlated with neurodevelopmental outcomes.

## Methods

### Data Availability Statement

Analytic methods underlying this article will be made available to other researchers on reasonable request and can be accessed by contacting the corresponding author. The data regarding participants will not be made available to other researchers due to privacy concerns. Further information about American Heart Association Journals’ data sharing policies can be found at https://www.ahajournals.org/data-sharing.

### Ethics Statement

This study protocol was reviewed and approved by the institutional review board of the Leiden University Medical Center (LUMC) with Approval No. 24-3042. As only retrospective and pseudonymized data were used, the need for written informed consent was waived by the institutional review board of the LUMC.

### Study Design and Participants

The LUMC is the national referral center in the Netherlands for complicated monochorionic twin pregnancies and fetal therapy. In this retrospective single-center observational cohort study, all monochorionic twins followed and those undergoing fetal therapy in our center, between January 2005 and December 2024, either with fetal demise or born at a gestational age (GA)<35 weeks, were eligible for the study. Infants were included if a diagnosis of PAIS was confirmed by neuroimaging—either cUS or MRI—before term-equivalent age (TEA; defined as 44 weeks’ corrected GA).

Per NICHD/NINDS guidelines (National Institute of Child Health and Human Development/National Institute of Neurological Disorders and Stroke), we excluded presumed PAIS—defined as stroke presenting and diagnosed >28 days postnatally (in preterms >44 weeks’ postmenstrual age)—to focus on cases with confirmed PAIS. Infants diagnosed with periventricular venous hemorrhagic infarction were excluded as this condition fell outside the scope of the study. All neuroimaging data were reevaluated by 2 experts in neonatal neurology (L.S.V. and S.J.S.) with >15 years’ experience in assessing cUS and MRI. Infants without neuroimaging scans available for confirmation were excluded from the study.

The institutional review board stated that this retrospective study did not apply to the Medical Research Involving Human Subjects Act (reference number 24-3042), and the need for informed consent was waived. To enhance research quality, reproducibility, and transparency, this study adhered to the STROBE guidelines (Strengthening the Reporting of Observational Studies in Epidemiology) for observational studies, with the corresponding checklist provided in Table S1.

### Clinical Data and Risk Factors

In the identified twin pregnancies with PAIS, clinical data were classified into maternal, fetal, placental, perinatal, and neonatal factors. The medical records of all eligible infants were systematically reviewed by 1 investigator (B.O.v.O.). When the twins were delivered outside the LUMC, missing clinical data were requested. For baseline characteristics, data during the whole hospital stay were collected for all twins, risk factors in the first week after birth, and neonatal period were collected up till the day of stroke diagnosis on neuroimaging.

### Twin Complications

Monochorionic pregnancies with PAIS were categorized as either uncomplicated or based on complications related to monochorionicity: (1) TTTS (treated with either complete or incomplete fetoscopic laser surgery (FLS), amniodrainage, or untreated); (2) spontaneous TAPS; (3) sFD, acute perimortem TTTS; (4) spontaneous monoamnionicity. TTTS was diagnosed and staged using standard prenatal ultrasound criteria.^18^ FLS is standard treatment at the LUMC in all TTTS cases presenting before 26 weeks’ gestation with TTTS >stage 1, or stage 1 with symptomatic polyhydramnios.^19^ Cases treated with FLS were considered complete if no recurrent TTTS or postlaser TAPS were observed. TAPS (RRID:SCR_002865) was diagnosed and staged antenatally according to Tollenaar et al.^20^ Spontaneous monoamnionicity was defined as the absence of the intertwin amniotic membrane and presence of only 1 yolk sac.^21^ A diagnosis of selective fetal growth restriction was defined as birth weight discordance within a twin pair >20%.^22^

### Neuroimaging

In our center, routine postnatal cUS was performed in all monochorionic twins with TTTS, TAPS, sFD, and selective fetal growth restriction; and those born before <32 weeks’ GA, with the first scan preferably between postnatal day 1 and 3. Additional cUS examinations were performed according to national protocol.^23^ Standard cUS images were obtained through the anterior and mastoid fontanelles.^24^ MRI was not performed routinely, but only in the presence of (suspected) ultrasound abnormalities. The MRI protocol involved T1-weighted and T2-weighted sequences in the coronal, sagittal, and transverse planes, with additional susceptibility-weighted imaging and diffusion-weighted imaging, and magnetic resonance angiography, if performed. MRI was performed on either a 1.5 Tesla or a 3 Tesla full-body Achieva system (Philips Medical Systems, Best, the Netherlands). Timing for MRI was determined on a case-by-case basis, depending on the clinical condition of the infant and following our institutional protocol for neonatal neuroimaging. In case of acute neurological symptoms or acute cUS changes, indicating a stroke of recent onset, MRI was performed preferably within 7 days, if the infant was medically stable, and included DWI, which can help to time the insult more accurately. For more chronic or established abnormalities, characterized by signs of atrophy or cystic evolution, an MRI was recommended at TEA. For all infants, MRI was performed, or repeated, at TEA to assess myelination in the corticospinal tract, and to validate, and if necessary, revise the stroke pattern classification, ensuring precise identification of the specific vascular territories. When arterial ischemic stroke (AIS) was classified as either antenatal or direct perinatal, available fetal ultrasound and fetal MRI scans were evaluated to allow for more precise timing.

Stroke patterns were classified on cUS based on the sonographic templates by Govaert^25^ and on MRI as described by Wagenaar et al.^26^ This classification was based on the shape, extent, and localization of the area of signal intensity change. In addition, involvement of deep gray matter (basal ganglia and thalamus), corticospinal tract (involvement of posterior limb of internal capsule and/or peduncle), central sulcus, punctate white matter lesions,^27^ presence of cortical sparing,^28^ and presence of intraventricular hemorrhage (classified according to Volpe^29^) were documented. Ventriculomegaly was defined as a ventricular index > 97th percentile according to Levene criteria on neonatal cUS.^30^

PAIS cases were allocated to 3 distinct groups based on the presumed timing of onset: (1) antenatal AIS (occurring in utero between 20 weeks’ gestation and onset of delivery), with either an antenatal diagnosis on abnormal fetal neuroimaging or cystic evolution/tissue loss detected on the first postnatal scan, performed ≤7 days after birth; (2) direct perinatal AIS (occurring between birth until the end of the first postnatal week), with acute changes (abnormal echogenicity visible on cUS or diffusion changes on MRI) detected ≤7 days after birth and often leading to cystic evolution or atrophy beyond 14 days after birth; (3) postnatal AIS (occurring after the first postnatal week and diagnosed before 44 weeks’ corrected GA). These infants did not have abnormalities on cUS imaging in the first week after birth, but later had imaging findings suggestive of AIS, often after a complicated course at the neonatal intensive care unit (NICU) and remote (at least >7 days) from the time of birth.

### Outcome

Data on symptoms at time of stroke diagnosis and neonatal mortality were collected. Among surviving infants, cerebral palsy was diagnosed during routine follow-up visits and its severity classified according to the Gross Motor Function Classification System (GMFCS).^25^ In cases where the GMFCS score was not available, we retrospectively assigned a GMFCS level based on the criteria established by Rosenbaum et al.^31^ Specifically, children who were able to walk without assistive devices were classified as GMFCS level I, therefore having mild cerebral palsy. If assessed, additional follow-up data on cognitive, fine, and gross motor development at 24 months corrected age were collected using the *Bayley Scales of Infant and Toddler Development, Third Edition*, or the *Griffiths Scales of Child Development, Second Edition*. Further data were collected on visual, auditory, and behavioral development, as well as the presence of epilepsy. Mild neurodevelopmental impairment (NDI) was defined as the presence of GMFCS level 1 cerebral palsy. Moderate-severe NDI was defined as the presence of cerebral palsy with GMFCS Level II or higher. The presence of cerebral visual impairment, epilepsy/hypsarrhythmia, and mortality (within the neonatal period or first year after birth) were classified as severe NDI.

### Statistical Analysis

The statistical analyses were conducted using SPSS Statistics version 29.0 (RRID:SCR_002865). Descriptive statistics were used to examine baseline and clinical characteristics and neurodevelopmental outcomes. Categorical data were presented as counts (percentage), whereas continuous data were presented as mean (95% CI) in case of normal distribution, or median (interquartile range) if the distribution of the data was skewed. To compare the 2 groups of monochorionic twins with stroke to their cotwins without stroke in terms of neonatal parameters and other variables, Fisher exact test was used for categorical data, and an unpaired *t* test was used for numerical data. A *P* <0.05 was considered statistically significant.

## Results

Among 1748 monochorionic twin pairs born from 2005 to 2024 and followed or treated at the LUMC, 1183 twin pairs were born at <35 weeks’ GA, in whom PAIS was diagnosed in 1 cotwin in 18 pairs (1.5%). No infants were excluded due to lack of neuroimaging. Clinical characteristics—including relevant obstetric, fetal, perinatal, neonatal, and placental factors—are listed in Table 1. The mean gestational age was 29.4 weeks (95% CI, 28.3–31.5), mean birth weight was 1258 g (95% CI, 1062–1453), 82% (14/17) were born in the LUMC, and 65% (11/17) were female. Median day of first scan with diagnosis of stroke was postnatal day 4 (1–12). Regarding the timing of onset, there were 6 infants with antenatal AIS, 7 infants with direct perinatal AIS, and 5 infants with postnatal AIS. Between these 3 groups, there was a shift from predominantly main branch middle cerebral artery (MCA) stroke patterns in the prenatal group, to predominantly smaller perforator strokes in the peri- and postnatal group (Table 1).

**Table 1.**
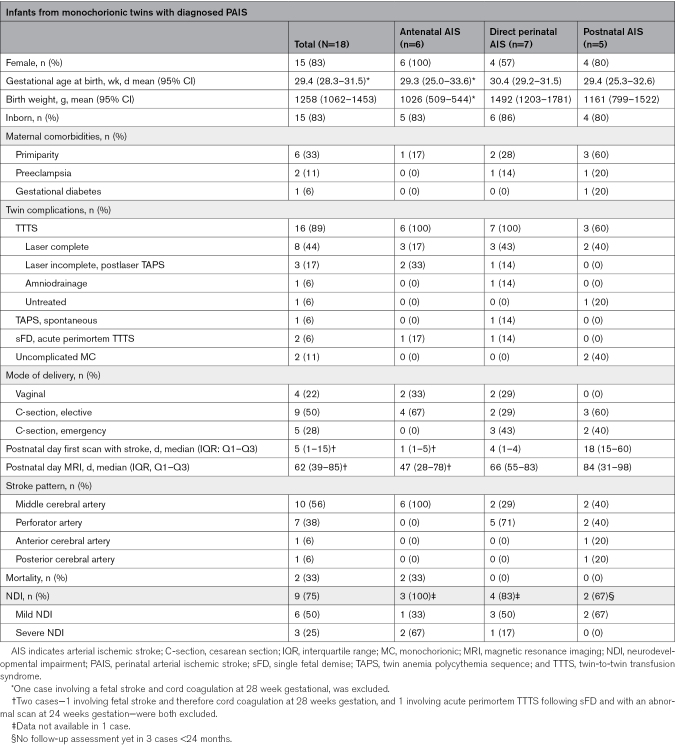
Baseline and Clinical Characteristics of Study Cohort

### Clinical Data and Risk Factors

Maternal and perinatal complications, along with neonatal parameters of infants with stroke compared with their unaffected cotwins, are summarized in Tables 2 and 3.

**Table 2.**
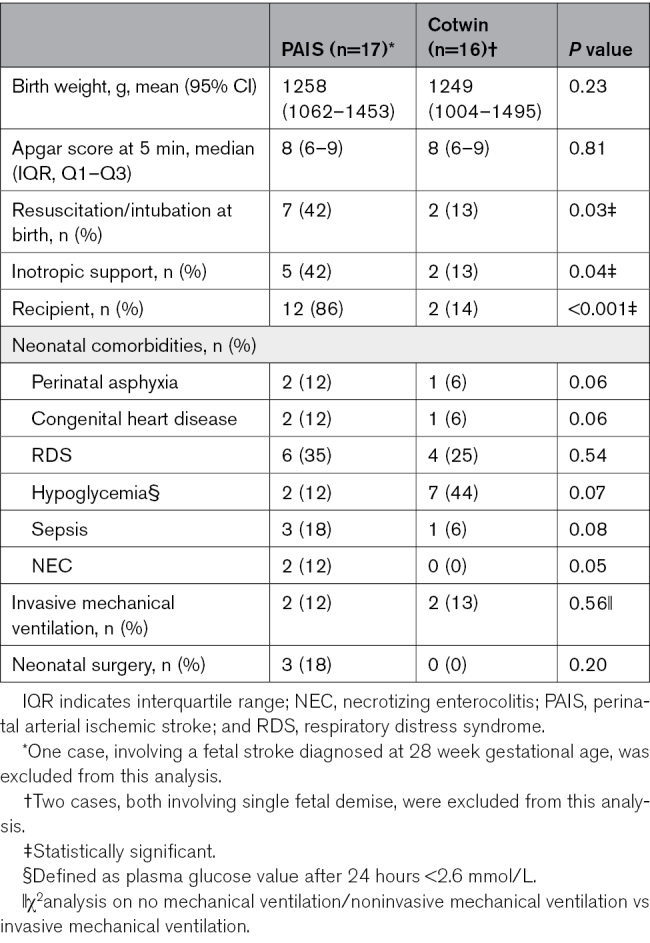
Clinical Characteristics Among Twins (Twin With PAIS Versus Unaffected Cotwin)

**Table 3.**
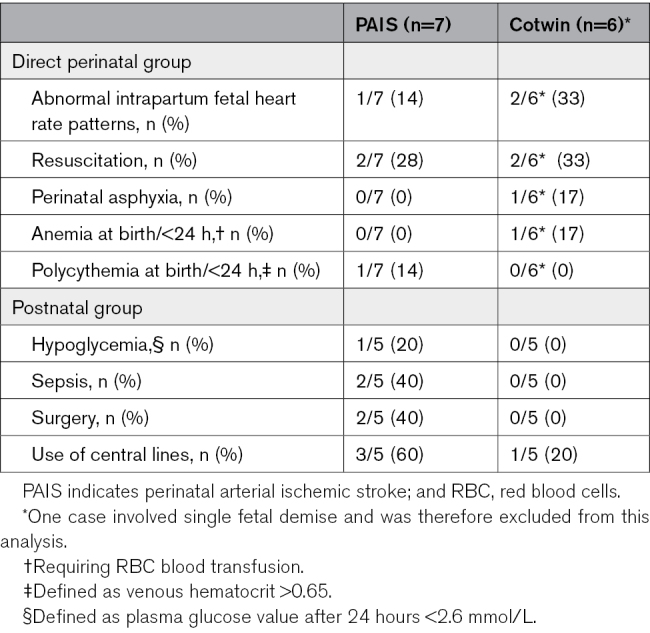
Identified Neonatal Risk Factors for PAIS (Twin With PAIS Versus Unaffected Cotwin)

Among the compared clinical characteristics, twins with PAIS did not differ significantly from their unaffected cotwin, except that in cases of TTTS/TAPS, significantly more (ex)recipient twins had PAIS as compared with donors (*P*<0.001; Table 2). In addition, significantly more infants with PAIS required resuscitation or intubation (*P*=0.03) or received inotropic support during hospital admission (*P*=0.04). Moreover, PAIS was diagnosed more often in infants with necrotizing enterocolitis (*P*=0.05). Regarding perinatal and neonatal risk factors, the groups were too small to perform statistical analysis; however, in the direct perinatal group, an emergency cesarean section was performed more frequently because of heart rate abnormalities in the cotwin without PAIS. In the postnatal group, sepsis, neonatal surgery, and the use of central lines were more common in the cotwins with PAIS. Risk factors are summarized in Table 3.

### Twin Complications

**Figure 1.**
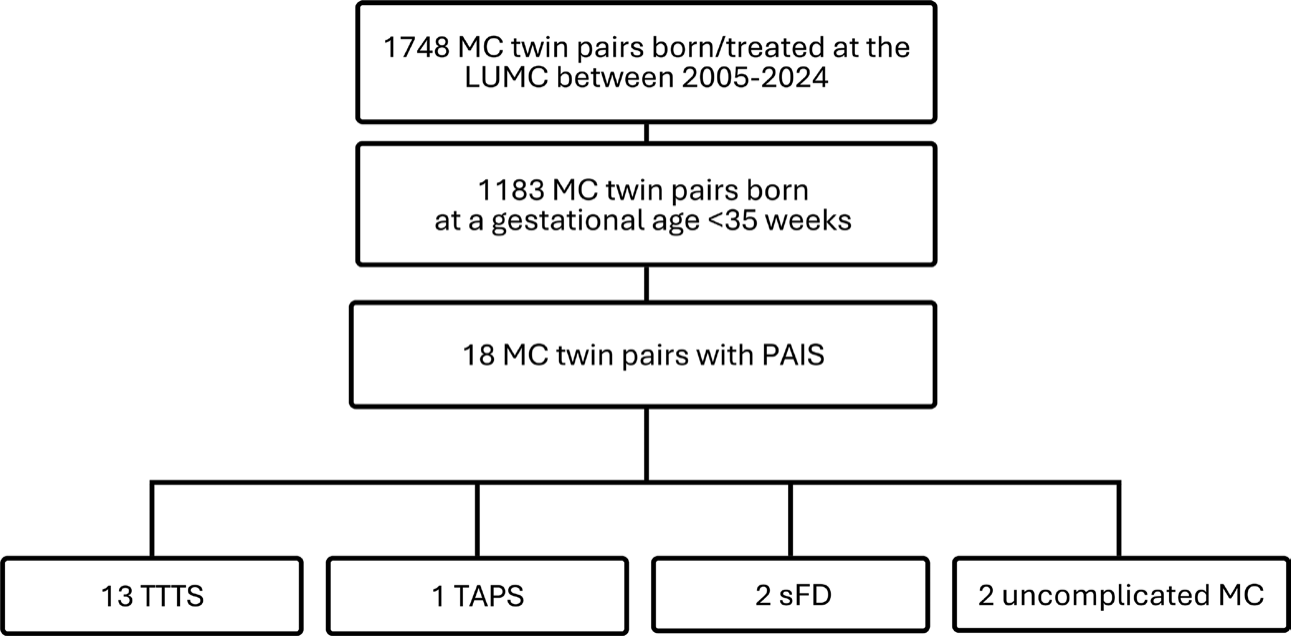
**Flowchart of monochorionic (MC) twin pregnancies followed/treated in Leiden University Medical Center (LUMC) and those complicated by perinatal arterial ischemic stroke (PAIS).** sFD indicates single fetal demise; TAPS, twin anemia polycythemia sequence; and TTTS, twin-to-twin transfusion syndrome.

Pregnancy complications related to monochorionicity were identified in 16 (89%) twin pairs; in the antenatal and direct perinatal AIS group, all pregnancies were complicated, whereas in the postnatal group, 2 twins did not experience any pregnancy complications related to monochorionicity. Complications included TTTS (n=13), TAPS (n=1), and sFD (n=2; Table 1; Figure 1). There were no cases of spontaneous monoamnionicity. Among the twins diagnosed with TTTS, 8 (62%) underwent complete FLS, and 3 (24%) developed postlaser TAPS after incomplete FLS. One of the postlaser TAPS pregnancies was treated with an intrauterine transfusion. In this case, due to the severe cerebral injury, a selective feticide was performed via umbilical cord coagulation at 28 weeks’ gestation (Table S2—twin 3).

### Stroke Patterns on Neuroimaging

**Figure 2.**
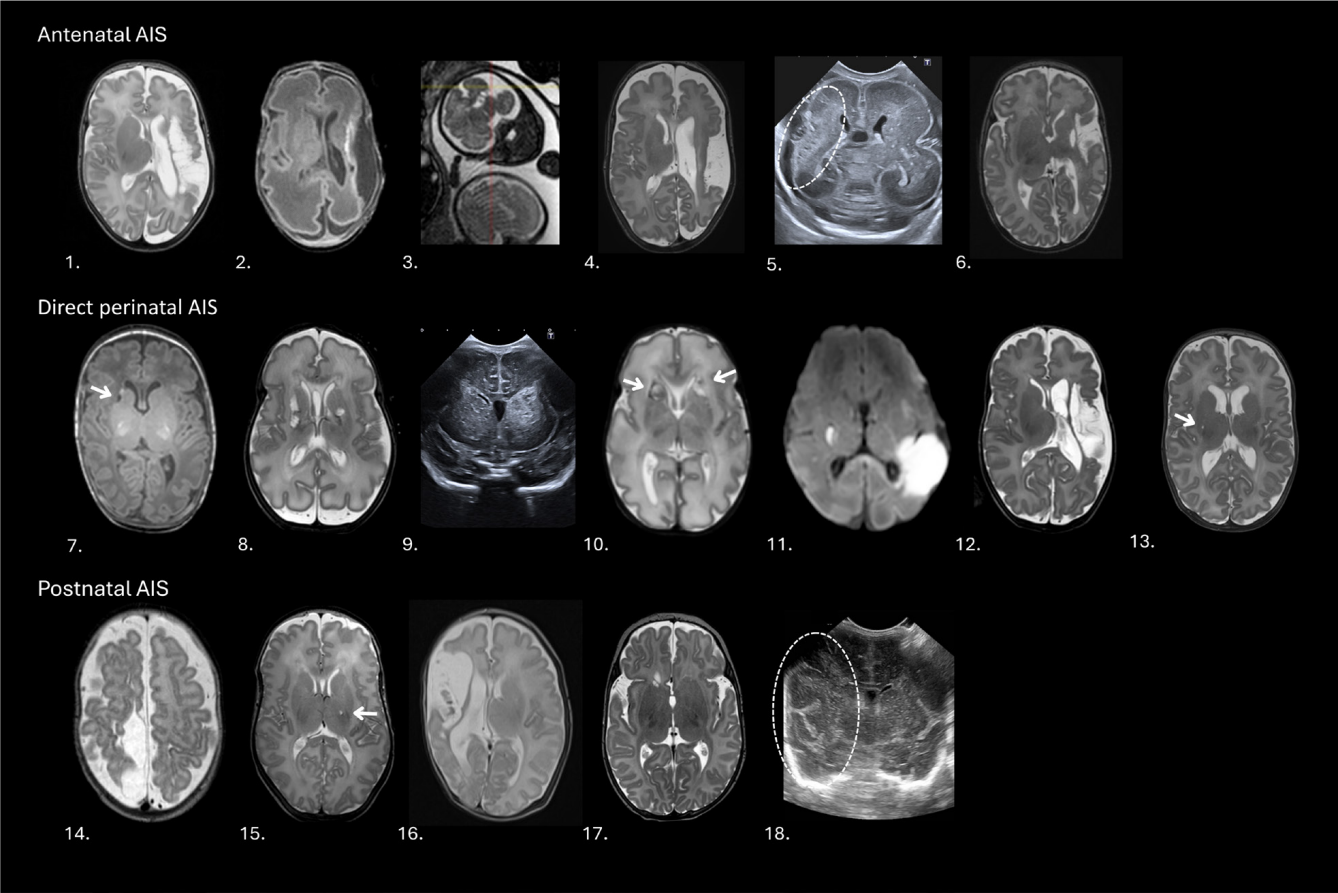
**Overview of all stroke patterns on neuroimaging, divided by presumed timing of perinatal arterial ischemic stroke (PAIS).** All magnetic resonance imaging (MRI) scans were performed at term-equivalent age, except for twin 3, who experienced a fetal stroke, leading to cord coagulation at 28 weeks’ gestation, and twin 5, who had an acute perimortem twin-to-twin transfusion syndrome following single fetal demise, with an abnormal scan at 24 weeks’ gestation. Both twins died in the neonatal period and therefore did not reach term-equivalent age to perform an MRI. Twin 11 underwent diffusion-weighted imaging in the first week after birth, showing diffusion restriction in both stroke territories.

Figure 2 provides an overview of stroke patterns in all monochorionic twins. Two cases were diagnosed on antenatal imaging (24 and 28 weeks’ GA; Table S2—twin 5; Table S3—twin 7); the remaining cases were diagnosed after birth. All but one of the infants had cUS as the initial neuroimaging technique. The median postnatal day of diagnostic cUS and confirmatory MRI with stroke were respectively 5 (interquartile range, 1–15) and 62 (interquartile range, 39–85). Stroke patterns observed included the MCA (anterior, posterior, or main branch) in 10 of 18 (56%); perforator arteries arising from MCA/anterior cerebral artery (ACA)/posterior communicating artery in 7 of 18 (39%); posterior cerebral artery in 1 of 18 (6%), and ACA in 1 of 18 (6%; Table 1). Two infants showed involvement of 2 stroke territories: 1 infant with a direct perinatal stroke of both a left posterior branch MCA and a right perforator MCA (Table S3—twin 11) and 1 infant with a postnatal stroke involving both the right main branch MCA and the right posterior cerebral artery (Table S4—twin 18).

### Antenatal AIS

**Figure 3.**
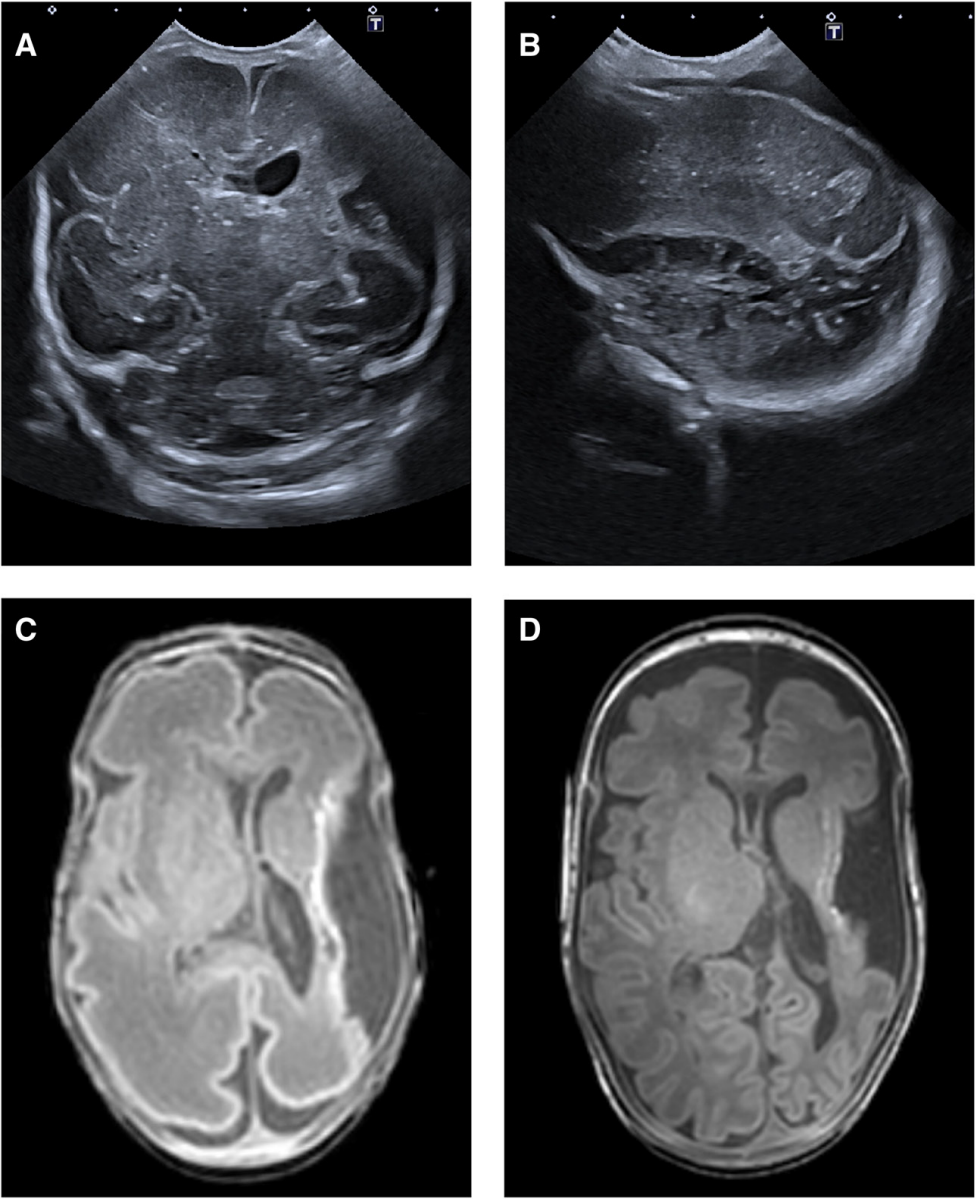
**Antenatal arterial ischemic stroke involving left main branch middle cerebral artery (MCA) in an ex-recipient twin with twin-to-twin transfusion syndrome, treated with complete fetoscopic laser surgery (twin 4). A**, Cranial ultrasound (cUS; coronal plane) at postnatal day 1 showing tissue loss in the left hemisphere and left lateral ventricle dilatation. **B**, cUS (sagittal plane) on the same day showing extensive cystic lesions within the parenchyma of the left hemisphere. **C**, Axial T1-weighted magnetic resonance imaging (MRI) at postnatal day 34 confirming a left main branch MCA (stroke) with ipsilateral ventriculomegaly, loss of cortex, and white matter abnormalities adjacent to operculum and insular region. **D**, Axial T2-weighted MRI at term-equivalent age demonstrating asymmetrical myelination of the posterior limb of the internal capsule. This infant subsequently developed unilateral spastic cerebral palsy (level 1) at 2 years of age (Table S2—twin 4).

All 6 infants with antenatal AIS had a stroke in the territory of the MCA (Table S2). Four of these 6 infants (67%) had a main branch MCA stroke (Figure 3), and 2 infants had an anterior branch MCA stroke. Four of the 6 cases (67%) were on the left side. In 4 infants, an MRI at TEA was performed, and showed involvement of the posterior limb of the internal capsule in 3 infants (the 2 surviving infants with a main branch MCA, and the 1 infant with anterior branch MCA stroke). One fetus and 1 infant died, and therefore an MRI could not be performed. In this group, both infants with anterior branch MCA stroke and 1 infant with main branch MCA stroke had cortical sparing (Table S2).

### Perinatal AIS

**Figure 4.**
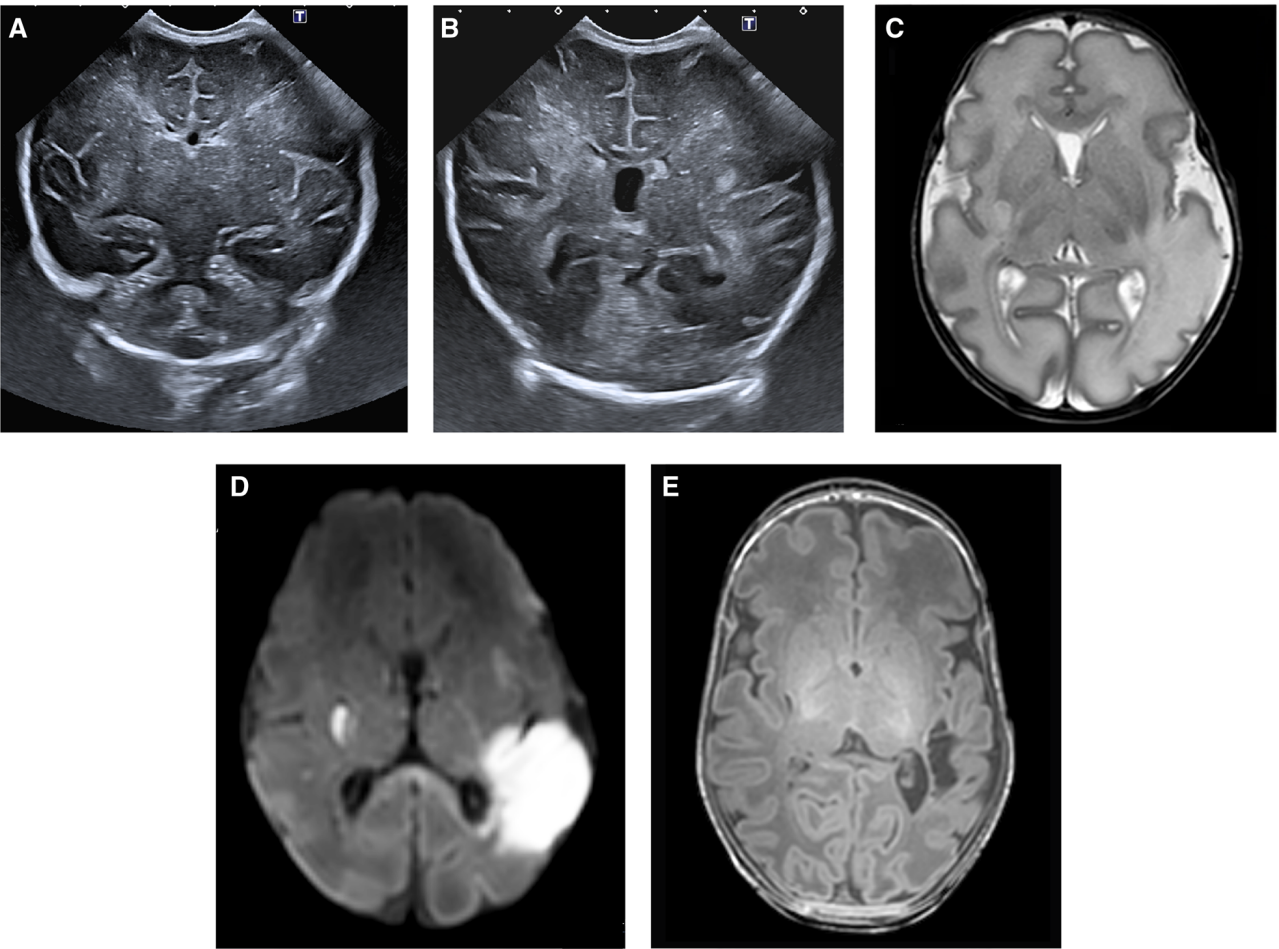
**Case of direct perinatal arterial ischemic stroke, involving posterior middle cerebral artery (MCA) stroke left and perforator stroke right, in an ex-recipient twin complicated by twin-to-twin transfusion syndrome (Q1) and treated with complete fetoscopic laser surgery (twin 11). A**, Cranial ultrasound (cUS) in coronal plane on postnatal day 4, revealing a small focus of increased echogenicity in the posterior right basal ganglia (encircled). **B**, cUS on the same day demonstrating bilateral white matter echogenicity, more pronounced on the left, extending towards the cortex. **C**, An MCA stroke was suspected, and therefore, a magnetic resonance imaging (MRI) on postnatal day 5 was indicated, showing on the axial T2-weighted image high signal intensity in the territory of the left posterior MCA branch, extending to the cortex, as well as the posterior right basal ganglia adjacent to the posterior limb of the internal capsule (PLIC). **D**, Diffusion-weighted imaging on the same day, showing diffusion restriction in both regions, confirming an acute stroke. **E**, A T1-weighted MRI at term-equivalent age demonstrating tissue loss in the posterior right basal ganglia and a small cyst in the right putamen and asymmetrical myelination of the PLIC, better on the left than right. The left-sided lesion in region of the posterior branch of the MCA had evolved into a cystic lesion adjacent to the left optic radiation, with white matter loss but relative preservation of the cortex. Clinically, the child developed early asymmetry of motor function, ultimately developing unilateral spastic cerebral palsy on the left (level 1). In addition, there is a risk of visual field asymmetry and later epilepsy (Table S3—twin 11).

Among the 7 infants with direct perinatal AIS, 4 (57%) had a stroke in the MCA territory, 1 involving the posterior branch, 1 the main branch and 2 smaller perforator (lenticulostriate arteries) strokes, of whom 1 infant showed a mixed pattern with both a left posterior branch MCA and a right perforator MCA stroke (Figure 4; Table S3—twin 11). The remaining 3 infants (43%) had a perforator stroke involving the ACA (Heubner artery), 2 unilateral, and 1 bilateral. All 7 infants underwent TEA-MRI. Involvement of the posterior limb of the internal capsule at TEA was seen in 3 infants (Table S3).

### Postnatal AIS

**Figure 5.**
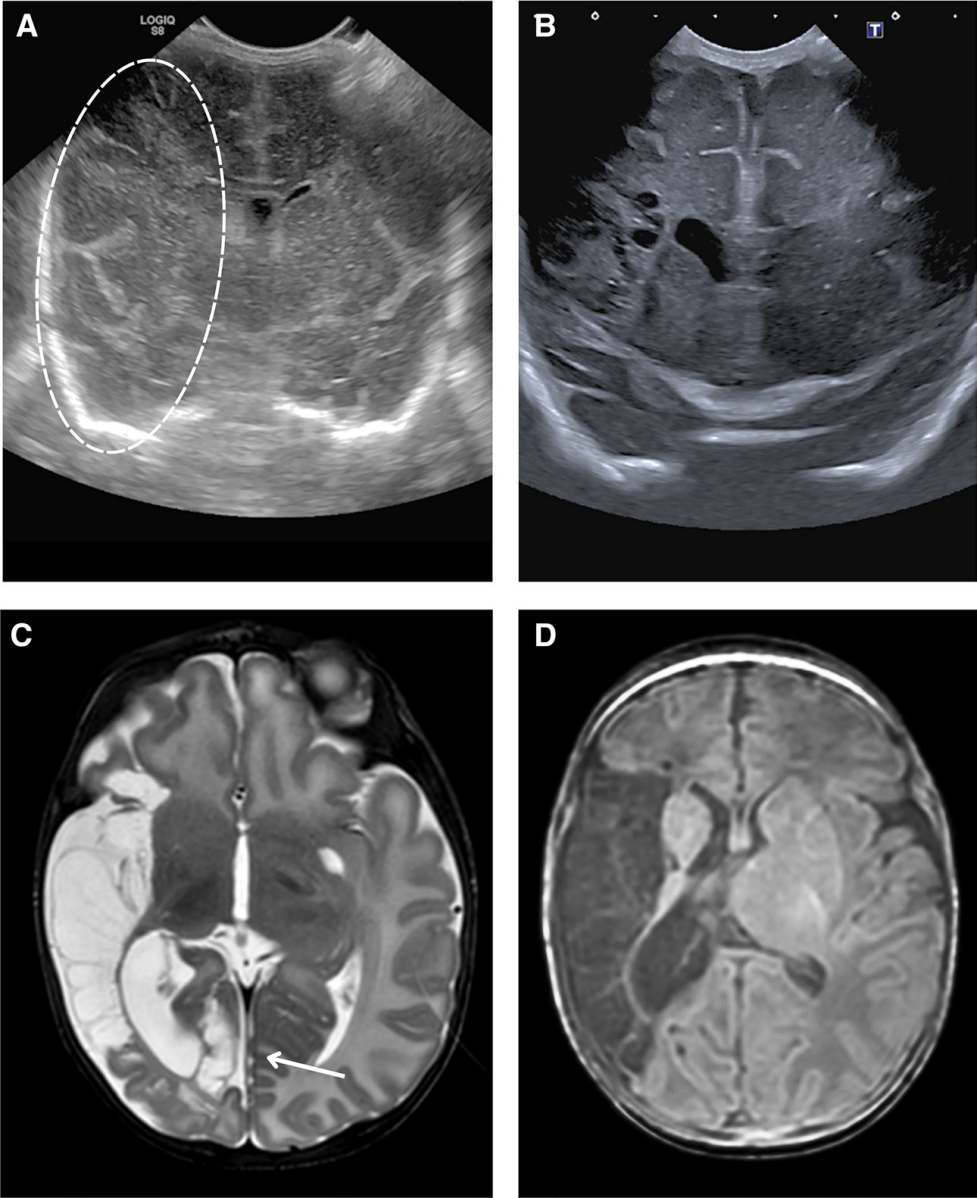
**Case of postnatal arterial ischemic stroke involving both right main branch middle cerebral artery (MCA) and posterior cerebral artery (PCA), in an uncomplicated twin pregnancy, with a second necrotizing enterocolitis surgery at postnatal 94 (twin 18). A**, Coronal cranial ultrasound (cUS) on postnatal day 95 demonstrating a demarcation line surrounding the right insula (encircled) and compression of the right lateral ventricle. **B**, Coronal cUS on postnatal day 134, revealing cystic lesions within the right hemisphere parenchyma and subsequent right lateral ventriculomegaly. **C**, Axial T2-weighted magnetic resonance imaging (MRI) at term-equivalent age confirming a right MCA territory stroke, also involving the PCA territory (arrow), with involvement of the right optic radiation. **D**, Axial T1-weighted MRI at the same time point demonstrating asymmetrical posterior limb of the internal capsule development. Although the infant was not yet seen at follow-up, these findings are consistent with a predicted outcome of left-sided hemiparesis, with potential sequelae including epilepsy and left-sided visual field deficits (Table S4—twin 18).

Among the 5 infants with postnatal onset of AIS, a mix of stroke patterns was observed. Two (40%) had a main branch MCA stroke, both occurring within 1 week after surgery for necrotizing enterocolitis (Figure 5; Table S4; twin 16 and twin 18). The other stroke patterns seen were a partial pial ACA (n=1), a posterior communicating artery perforator (n=1), and an ACA perforator (n=1) stroke. The posterior limb of the internal capsule was involved in 3 infants, including the 2 main branch MCA strokes.

### Additional Involvement

Other cranial lesions observed in the study population included intraventricular hemorrhage (intraventricular hemorrhage grade 1 in 5/18 [28%]; intraventricular hemorrhage grade 3 in 1/18 [6%]) and punctate white matter lesions (3/18; 11%). Involvement of the optic radiation was seen in 3 infants.

### Outcome

All infants were asymptomatic at the time of PAIS diagnosis. One infant with an MCA stroke of antenatal onset died in the neonatal period following redirection of care (Table S2—twin 5). Of the 16 survivors, follow-up at 2 years was available in 12 infants, with 3 still too young to be assessed, and 1 lost to follow-up. Six showed mild NDI (50%) and 3 (25%) severe NDI (Table S2). One of these 3 with an antenatal AIS developed hypsarrhythmia (Table S2—twin 1), 1 also with an antenatal AIS developed cerebral visual impairment (Tables S2 through S6), and 1 infant with a direct perinatal AIS developed focal epilepsy (Table S3—twin 12).

In the antenatal AIS group, 2 of the 3 surviving infants with known follow-up developed unilateral spastic cerebral palsy, and both showed evidence of severe NDI. In the direct perinatal AIS group, 2 infants exhibited normal development, 2 presented with mild unilateral spastic cerebral palsy, and 1 developed focal epilepsy. In this group, 1 infant with bilateral perforator ACA stroke exhibited no motor impairment, but showed mild cognitive and behavioral impairment as indicated by low scores on the *Bayley Scales of Infant and Toddler Development, Third Edition* and *Child Behavior Checklist* (CBCL; <1 SD; Table S3—twin 10). Two infants with postnatal AIS had not yet reached 2 years of age. Among those with postnatal AIS onset and available follow-up data, 2 had mild unilateral spastic cerebral palsy, whereas 1 exhibited no motor impairment.

## Discussion

This study is the first, to our knowledge, to specifically investigate the incidence and timing of PAIS in preterm monochorionic twins and demonstrates that these twins are at risk of PAIS, not only antenatally but also in the direct perinatal and postnatal period.

Among 1183 twin pairs born <35 weeks GA, 18 had PAIS in 1 twin, resulting in an overall incidence of 1.5% in monochorionic pregnancies treated or delivered in our hospital and 0.8% among all monochorionic twins born <35 weeks. We only included infants born <35 weeks in our study, as the majority of these infants are admitted to a NICU or high care neonatal unit and will have at least 1 routine cUS within the first week after birth. Moreover, most monochorionic twins, especially when complicated, are born before 35 weeks.^32^ The incidence aligns with a previously reported incidence of PAIS of 0.7% in preterm infants (GA <35 weeks) admitted to a NICU in the Netherlands^8^ but is much higher than the 0.09% reported in a nationwide German study (87 per 100.000 preterm births <34 weeks GA).^33^ Notably, not all twins in our study were admitted to our NICU. Of the 18 cases with PAIS, 15 (83%) were inborn, and 3 were admitted to another Dutch NICU. Consequently, PAIS cases born in other centers and not admitted to a NICU may have undergone less strict cUS screening, potentially leading to an underestimation of PAIS in the total monochorionic twin pair population.

Risk factor analysis showed no significant trends, likely due to the small sample size, except for recipient status, which was significantly associated with PAIS in all 3 time groups (*P*<0.001). Neuroimaging and outcome were different between the 3 groups. The antenatal AIS group exhibited the most extensive stroke patterns, correlating with death or severe NDI. The direct perinatal AIS group showed predominantly smaller perforator strokes, and infants in this group showed either no or mild NDI. All pregnancies in these 2 groups had complications related to monochorionicity. In contrast, the postnatal AIS group was heterogeneous in terms of pregnancy complications, donor/recipient status, stroke patterns, and outcomes. Notably, 2 twins in this group developed main branch MCA stroke following surgery for necrotizing enterocolitis later in the neonatal period.

Overall, 16 twin pairs experienced monochorionicity-related pregnancy complications, predominantly TTTS (71%). This finding aligns with prior studies identifying TTTS as a major risk factor for PAIS in preterm neonates.^9^

Several potential mechanisms have been proposed for antenatal AIS in twin pregnancies, with recipient twins being particularly susceptible, consistent with our findings.^14^ One potential mechanism is placental embolism—triggered by laser therapy or occurring at the end of gestation—allowing emboli to enter the fetal circulation and reach the cerebral circulation via the patent foramen ovale.^5,34^ In addition, Miller^34^ suggested that recipient twins may develop polycythemia and hyperviscosity, exacerbating cerebral injury in preterm infants with immature vasculature.^34^ Air embolism during cesarean section, a common delivery mode in twins (78% in our cohort), may also contribute to cerebral infarction. Despite these hypotheses, the precise pathophysiology of PAIS in recipient twins remains unclear.

Lopriore et al^35^ suggested that untreated or incompletely treated TTTS twins have an increased risk for injury and mortality. Incomplete laser therapy, leading to postlaser TAPS, may increase the risk of cerebrovascular injury because the twins are experiencing a second hit of hemodynamic instability. However, the exact timing of cerebral injury in TTTS remains unclear, as injury may occur before, during, or after FLS.^36^ Prospective studies with serial fetal neuroimaging are therefore needed to better understand the timing and cause of AIS in the antenatal period.

In the direct perinatal AIS group, it has been hypothesized that recipients who are still recovering from intervention and hemodynamic changes after FLS may experience further fluctuations in cerebral vascular flow during delivery. Whether this second hit phenomenon may lead to a condition like PAIS has yet to be confirmed. Although abnormal intrapartum fetal heart rate patterns have been associated with PAIS in preterm infants,^9^ this was not observed in our cohort.

For postnatal AIS, known risk factors in singleton preterm infants for PAIS include neonatal hypoglycemia,^9,37,38^ sepsis,^39^ and vascular catheter-related thrombosis. These were more prevalent in our postnatal AIS group; however, the small sample size precluded statistical analysis. Therefore, we recommend larger, multicenter studies with more substantial study populations to draw definitive conclusions regarding risk factors.

Interestingly, our results showed a female predominance, contrasting with the male predominance typically observed in term infants with perinatal stroke.^1,40^ This also differs from Sorg et al,^33^ who reported male predominance in preterm infants, but aligns with Benders et al,^8^ who found no sex-based differences in their population.

The strength of this study lies in its access to a large, closely monitored cohort of monochorionic twin pregnancies with systematically collected data on pregnancy, delivery, and postnatal complications in the Dutch national referral center for complicated monochorionic twin pregnancies and fetal therapy. However, its retrospective design presents limitations. First, accurately defining the onset of stroke is challenging when relying on existing neuroimaging data. To address the distinction between direct perinatal and postnatal AIS, we chose a 7-day cutoff, as most strokes with an onset around the time of birth will become sonographically apparent within the first week after birth. Detection rates rise significantly after day 4 and reach ≈87% by day 7, as supported by previous studies in full-term infants.^41,42^ We acknowledge that making this distinction purely on retrospective cUS can be challenging for some cases. For example, 1 infant (twin 13) presented with stroke-related abnormalities on day 7, despite a normal ultrasound on day 3. Because it is unclear whether the stroke developed after the initial scan or was undetectable at that time, the case was classified as direct perinatal AIS. Another case (twin 14) showed cystic evolution on day 17, with no prior scans indicating acute changes during the first week. This infant had been transferred to another hospital on day 4, and no imaging was performed between day 2 and day 17, making it impossible to estimate precisely the timing of stroke onset. Regarding cystic evolution or tissue loss, we used a >14-day threshold after birth or after the onset of echogenicity, based on literature indicating that cystic changes typically develop between 2 weeks and 1 month postinjury.^43^ We recognize the need to refine these definitions and aim to address this in future prospective studies involving larger cohorts.

Defining the timing of stroke onset is further complicated by long prenatal screening intervals, which limit the precision in timing of antenatal AIS or its association with fetal therapy. In addition, not all pregnancies resulted in delivery at our center, leading to variability in postnatal imaging and potential underdiagnosis at other institutions. Routine postnatal cUS is not performed in all infants, particularly those born after 34 weeks, further increasing the likelihood of missed diagnoses. Smaller perforator strokes, often asymptomatic, are especially prone to being overlooked without serial cUS. To improve detection and recognition of cases that may otherwise present as presumed PAIS, future studies should assess the value of prospectively performed routine postnatal cUS at the end of the first week in all monochorionic twins, especially those with complications due to monochorionicity. Finally, the small sample size of follow-up data restricts conclusions on neurodevelopmental outcomes.

In conclusion, although most research has focused on the antenatal mechanisms of stroke in monochorionic twins, this study demonstrated that PAIS can also occur in the direct perinatal and postnatal period. Early and routine fetal neuroimaging, particularly in recipients of fetal therapy, is essential for detecting antenatal AIS. Postnatally, serial cUS in the first week is crucial for identifying smaller, often asymptomatic perinatal AIS. In preterm infants admitted to the NICU, routine ultrasound screening, especially following neonatal risk factors such as central line removal and surgery, facilitates detection of postnatal AIS. Further prospective research is needed to determine the precise incidence, timing, and long-term impact of PAIS in monochorionic twins.

## Article Information

### Acknowledgments

The authors express their sincere gratitude to the Division of Neonatology, Department of Pediatrics, Leiden University Medical Center, for their collaboration and flexibility, which enabled them to conduct this single-center retrospective cohort study. Moreover, they are deeply thankful to all the participants whose involvement made this research possible.

### Author Contributions

B.O. van Oldenmark conceptualized and designed the study, collected data, assessed neuroimaging, drafted the initial manuscript, and critically reviewed and revised the manuscript. Dr Rondagh collected data, critically reviewed, and revised the manuscript. Dr Adama van Scheltema reevaluated available fetal neuroimaging. Dr Slaghekke contributed data and critically reviewed and revised the manuscript. Dr van der Meeren critically reviewed and revised the manuscript. Dr Spruijt contributed data and critically reviewed and revised the manuscript. Dr Lopriore conceptualized and designed the study, collected data, critically reviewed, and revised the manuscript. Dr de Vries conceptualized and designed the study, collected data, assessed neuroimaging, critically reviewed, and revised the manuscript. Dr Steggerda conceptualized and designed the study, collected data, assessed neuroimaging, critically reviewed, and revised the manuscript.

### Sources of Funding

This study received funding from the Cerebral Palsy Alliance (Australia) and the Phelps Foundation (the Netherlands). They did not take part in designing this study.

### Disclosures

None.

### Supplemental Material

Table S1: STROBE Checklist

Tables S2–S4

Figures S1–S3

## References

[1] Chabrier S Husson B Dinomais M Landrieu P Nguyen The Tich S. New insights (and new interrogations) in perinatal arterial ischemic stroke. Thromb Res. 2011;127:13–22. doi: 10.1016/j.thromres.2010.10.003 21055794 10.1016/j.thromres.2010.10.003

[2] Raju TN Nelson KB Ferriero D Lynch JK; NICHD-NINDS Perinatal Stroke Workshop Participants. Ischemic perinatal stroke: summary of a workshop sponsored by the National Institute of Child Health and Human Development and the National Institute of Neurological Disorders and Stroke. Pediatrics. 2007;120:609–616. doi: 10.1542/peds.2007-0336 17766535 10.1542/peds.2007-0336

[3] Oskoui M Shevell MI. Profile of pediatric hemiparesis. J Child Neurol. 2005;20:471–476. doi: 10.1177/088307380502000601 15996394 10.1177/088307380502000601

[4] Calandrino A Cipresso G Battaglini M Caruggi S Bonato I Massirio P Andreato C Vinci F Parodi A Malova M. Neonatal perforator stroke: timing, risk factors, and neurological outcome from a single-center experience. Neurol Int. 2025;17:59. doi: 10.3390/neurolint17040059 40278430 10.3390/neurolint17040059 PMC12029835

[5] Elbers J Viero S MacGregor D DeVeber G Moore AM. Placental pathology in neonatal stroke. Pediatrics. 2011;127:e722–e729. doi: 10.1542/peds.2010-1490 21339263 10.1542/peds.2010-1490

[6] Whitaker EE Cipolla MJ. Perinatal stroke. Handb Clin Neurol. 2020;171:313–326. doi: 10.1016/B978-0-444-64239-4.00016-3 32736758 10.1016/B978-0-444-64239-4.00016-3

[7] Armstrong-Wells J Ferriero DM. Diagnosis and acute management of perinatal arterial ischemic stroke. Neurol Clin Pract. 2014;4:378–385. doi: 10.1212/CPJ.0000000000000077 25317375 10.1212/CPJ.0000000000000077 PMC4196460

[8] Benders MJ Groenendaal F Uiterwaal CS de Vries LS. Perinatal arterial stroke in the preterm infant. Semin Perinatol. 2008;32:344–349. doi: 10.1053/j.semperi.2008.07.003 18929157 10.1053/j.semperi.2008.07.003

[9] Benders MJ Groenendaal F Uiterwaal CS Nikkels PG Bruinse HW Nievelstein RA de Vries LS. Maternal and infant characteristics associated with perinatal arterial stroke in the preterm infant. Stroke. 2007;38:1759–1765. doi: 10.1161/STROKEAHA.106.479311 17495219 10.1161/STROKEAHA.106.479311

[10] Grether JK Nelson KB Cummins SK. Twinning and cerebral palsy: experience in four northern California counties, births 1983 through 1985. Pediatrics. 1993;92:854–858. doi: 8233749

[11] Petterson B Nelson KB Watson L Stanley F. Twins, triplets, and cerebral palsy in births in Western Australia in the 1980s. BMJ. 1993;307:1239–1243. doi: 10.1136/bmj.307.6914.1239 8281055 10.1136/bmj.307.6914.1239 PMC1679356

[12] Pharoah P. Neurological outcome in twins. Semin Neonatol. 2002;7:223–230. doi: 10.1053/siny.2002.0109 12234746 10.1053/siny.2002.0109

[13] Benirschke K. The biology of the twinning process: how placentation influences outcome. Semin Perinatol. 1995;19:342–350. doi: 10.1016/s0146-0005(05)80012-6 8821022 10.1016/s0146-0005(05)80012-6

[14] Spruijt M Steggerda S Rath M van Zwet E Oepkes D Walther F Lopriore E. Cerebral injury in twin-twin transfusion syndrome treated with fetoscopic laser surgery. Obstet Gynecol. 2012;120:15–20. doi: 10.1097/AOG.0b013e31825b9841 22914388 10.1097/AOG.0b013e31825b9841

[15] Tricca S Parazzini C Doneda C Arrigoni F Tortora M Lanna M Casati D Faiola S Righini A Izzo G. Magnetic resonance imaging of intracranial anomalies in pregnancies complicated by twin anemia-polycythemia sequence. Neuroradiology. 2024;66:1213–1223. doi: 10.1007/s00234-024-03373-4 38720066 10.1007/s00234-024-03373-4 PMC11150324

[16] El Emrani S Groene SG Verweij EJ Slaghekke F Khalil A van Klink JMM Tiblad E Lewi L Lopriore E. Gestational age at birth and outcome in monochorionic twins with different types of selective fetal growth restriction: a systematic literature review. Prenat Diagn. 2022;42:1094–1110. doi: 10.1002/pd.6206 35808908 10.1002/pd.6206 PMC9543733

[17] Kirton A Armstrong-Wells J Chang T Deveber G Rivkin MJ Hernandez M Carpenter J Yager JY Lynch JK Ferriero DM; International Pediatric Stroke Study Investigators. Symptomatic neonatal arterial ischemic stroke: the International Pediatric Stroke Study. Pediatrics. 2011;128:e1402–e1410. doi: 10.1542/peds.2011-1148 22123886 10.1542/peds.2011-1148

[18] Quintero RA Morales WJ Allen MH Bornick PW Johnson PK Kruger M. Staging of twin-twin transfusion syndrome. J Perinatol. 1999;19:550–555. doi: 10.1038/sj.jp.7200292 10645517 10.1038/sj.jp.7200292

[19] Lopriore S. Gecompliceerde monochoriale tweelingen (TTS, TAPS, sFGR). NICU Neonatologie (Version 7). LUMC 2020. Accessed December 22, 2024. https://www.lumc.nl/siteassets/over-het-lumc/afdelingen/neonatologie/bestanden/gecompliceerde-monochoriale-tweelingen.pdf?HyperlinkID=fdeea9bd-056a-46f9-ab46-44f5745e75c8

[20] Tollenaar LSA Lopriore E Middeldorp JM Haak MC Klumper FJ Oepkes D Slaghekke F. Improved prediction of twin anemia-polycythemia sequence by delta middle cerebral artery peak systolic velocity: new antenatal classification system. Ultrasound Obstet Gynecol. 2019;53:788–793. doi: 10.1002/uog.20096 30125414 10.1002/uog.20096 PMC6593803

[21] Khairudin D Khalil A. Monochorionic monoamniotic twin pregnancies. Best Pract Res Clin Obstet Gynaecol. 2022;84:96–103. doi: 10.1016/j.bpobgyn.2022.08.004 36123247 10.1016/j.bpobgyn.2022.08.004

[22] Groene SG Stegmeijer KJJ Tan RNGB Steggerda SJ Haak MC Slaghekke F Roest AAW Heijmans BT Lopriore E van Klink JMM. Long-term effects of selective fetal growth restriction (LEMON): a cohort study of neurodevelopmental outcome in growth discordant identical twins in the Netherlands. Lancet Child Adolesc Health. 2022;6:624–632. doi: 10.1016/S2352-4642(22)00159-6 35871831 10.1016/S2352-4642(22)00159-6

[23] Leijser LMMG Mulder-de Tollenaer SM. Landelijke aanbeveling—Neonatale Neuro-imaging (versie 1.5). 2015. Accessed December 22, 2024. https://neonatology.eu/sites/default/files/neonatale_neuroimaging_versie_1_5_feb_2015.pdf

[24] Meijler G. Neonatal Cranial Ultrasonography. Springer Berlin Heidelberg; 2012.

[25] Govaert P. Sonographic stroke templates. Semin Fetal Neonatal Med. 2009;14:284–298. doi: 10.1016/j.siny.2009.07.006 19682961 10.1016/j.siny.2009.07.006

[26] Wagenaar N Martinez-Biarge M van der Aa NE van Haastert IC Groenendaal F Benders M Cowan FM de Vries LS. Neurodevelopment after perinatal arterial ischemic stroke. Pediatrics. 2018;142:e20174164. doi: 10.1542/peds.2017-4164 30072575 10.1542/peds.2017-4164

[27] Kersbergen KJ Benders MJ Groenendaal F Koopman-Esseboom C Nievelstein RA van Haastert IC de Vries LS. Different patterns of punctate white matter lesions in serially scanned preterm infants. PLoS One. 2014;9:e108904. doi: 10.1371/journal.pone.0108904 25279755 10.1371/journal.pone.0108904 PMC4184838

[28] van der Aa NE Benders MJ Nikkels PG Groenendaal F de Vries LS. Cortical sparing in preterm ischemic arterial stroke. Stroke. 2016;47:869–871. doi: 10.1161/STROKEAHA.115.011605 26757751 10.1161/STROKEAHA.115.011605

[29] Inder TE Perlman JM Volpe JJ. Chapter 24—preterm intraventricular hemorrhage/posthemorrhagic hydrocephalus. In: Volpe JJ Inder TE Darras BT de Vries LS du Plessis AJ Neil JJ Perlman JM eds. Volpe’s Neurology of the Newborn (Sixth Edition). Elsevier; 2018:637.e621–698.e621.

[30] Levene MI. Measurement of the growth of the lateral ventricles in preterm infants with real-time ultrasound. Arch Dis Child. 1981;56:900–904. doi: 10.1136/adc.56.12.900 7332336 10.1136/adc.56.12.900 PMC1627506

[31] Rosenbaum PL Walter SD Hanna SE Palisano RJ Russell DJ Raina P Wood E Bartlett DJ Galuppi BE. Prognosis for gross motor function in cerebral palsy: creation of motor development curves. JAMA. 2002;288:1357–1363. doi: 10.1001/jama.288.11.1357 12234229 10.1001/jama.288.11.1357

[32] Seetho S Kongwattanakul K Saksiriwuttho P Thepsuthammarat K. Epidemiology and factors associated with preterm births in multiple pregnancy: a retrospective cohort study. BMC Pregnancy Childbirth. 2023;23:872. doi: 10.1186/s12884-023-06186-0 38110899 10.1186/s12884-023-06186-0 PMC10726547

[33] Sorg AL Von Kries R Klemme M Gerstl L Beyerlein A Lack N Felderhoff-Müser U Dzietko M. Incidence and risk factors of cerebral sinovenous thrombosis in infants. Dev Med Child Neurol. 2021;63:697–704. doi: 10.1111/dmcn.14816 33506500 10.1111/dmcn.14816

[34] Miller V. Neonatal cerebral infarction. Semin Pediatr Neurol. 2000;7:278–288. doi: 10.1053/spen.2000.20076 11205717 10.1053/spen.2000.20076

[35] Lopriore EA Slaghekke F Verweij EJ Haak MC Middeldorp AJM Lopriore E. Neonatal outcome in twin-to-twin transfusion syndrome not treated with fetoscopic laser surgery. Twin Res Hum Genet. 2022;25:45–49. doi: 10.1017/thg.2022.5 35644997 10.1017/thg.2022.5

[36] Banek CS Hecher K Hackeloer BJ Bartmann P. Long-term neurodevelopmental outcome after intrauterine laser treatment for severe twin-twin transfusion syndrome. Am J Obstet Gynecol. 2003;188:876–880. doi: 10.1067/mob.2003.202 12712079 10.1067/mob.2003.202

[37] Ecury-Goossen GM Raets MM Lequin M Feijen-Roon M Govaert P Dudink J. Risk factors, clinical presentation, and neuroimaging findings of neonatal perforator stroke. Stroke. 2013;44:2115–2120. doi: 10.1161/STROKEAHA.113.001064 23723310 10.1161/STROKEAHA.113.001064

[38] Golomb MR Garg BP Edwards-Brown M Williams LS. Very early arterial ischemic stroke in premature infants. Pediatr Neurol. 2008;38:329–334. doi: 10.1016/j.pediatrneurol.2007.12.012 18410848 10.1016/j.pediatrneurol.2007.12.012 PMC2770811

[39] Sorg AL von Kries R Klemme M Gerstl L Felderhoff-Müser U Dzietko M. Incidence estimates of perinatal arterial ischemic stroke in preterm- and term-born infants: a national capture-recapture calculation corrected surveillance study. Neonatology. 2021;118:727–733. doi: 10.1159/000514922 33794541 10.1159/000514922

[40] Golomb MR MacGregor DL Domi T Armstrong DC McCrindle BW Mayank S deVeber GA. Presumed pre- or perinatal arterial ischemic stroke: risk factors and outcomes. Ann Neurol. 2001;50:163–168. doi: 10.1002/ana.1078 11506398 10.1002/ana.1078

[41] Cowan F Mercuri E Groenendaal F Bassi L Ricci D Rutherford M de Vries L. Does cranial ultrasound imaging identify arterial cerebral infarction in term neonates? Arch Dis Child Fetal Neonatal Ed. 2005;90:F252–F256. doi: 10.1136/adc.2004.055558 15846018 10.1136/adc.2004.055558 PMC1721893

[42] Olivé G Agut T Echeverría-Palacio CM Arca G García-Alix A. Usefulness of cranial ultrasound for detecting neonatal middle cerebral artery stroke. Ultrasound Med Biol. 2019;45:885–890. doi: 10.1016/j.ultrasmedbio.2018.11.004 30642660 10.1016/j.ultrasmedbio.2018.11.004

[43] Dudink J Mercuri E Al-Nakib L Govaert P Counsell SJ Rutherford MA Cowan FM. Evolution of unilateral perinatal arterial ischemic stroke on conventional and diffusion-weighted MR imaging. AJNR Am J Neuroradiol. 2009;30:998–1004. doi: 10.3174/ajnr.A1480 19193752 10.3174/ajnr.A1480 PMC7051645

